# The Meaning of Emoji to Describe Food Experiences in Pre-Adolescents

**DOI:** 10.3390/foods9091307

**Published:** 2020-09-16

**Authors:** Julia Sick, Erminio Monteleone, Lapo Pierguidi, Gastón Ares, Sara Spinelli

**Affiliations:** 1 Department of Agriculture, Food, Environment and Forestry (DAGRI), University of Florence, Via Donizetti 6, 50144 Florence, Italy; erminio.monteleone@unifi.it (E.M.); lapo.pierguidi@unifi.it (L.P.); sara.spinelli@unifi.it (S.S.); 2 Sensometrics and Consumer Science, Instituto Polo Tecnológico de Pando, Facultad de Química, Universidad de la República, Pando, 91000 Canelones, Uruguay; gares@fq.edu.uy

**Keywords:** pre-adolescents, emotions, arousal, power, emoji, food, projective mapping, emotion words

## Abstract

Ongoing research has shown that emoji can be used by children to discriminate food products, but it is unclear if they express emotions and how they are linked to emotional words. Little is known about how children interpret emoji in terms of their emotional meaning in the context of food. This study aimed at investigating the emotional meaning of emoji used to describe food experiences in 9–13-year-old pre-adolescents and to measure related age and gender differences. The meaning of 46 emoji used to describe food experience was explored by: mapping emoji according to similarities and differences in their emotional meaning using the projective mapping technique, and linking emoji with emotion words using a check-all-that-apply (CATA) format. The two tasks gave consistent results and showed that emoji were discriminated along the valence (positive vs. negative) and power (dominant vs. submissive) dimension, and to a lower extent along the arousal dimension (high vs. low activation). In general, negative emoji had more distinct meanings than positive emoji in both studies, but differences in nuances of meaning were found also among positive emoji. Girls and older pre-adolescents (12–13 years old (y.o.)) discriminated positive emoji slightly better than boys and younger pre-adolescents (9–11 y.o.). This suggests that girls and older pre-adolescents may be higher in emotional granularity (the ability to experience and discriminate emotions), particularly of positive emotions. The results of the present work can be used for the development of an emoji-based tool to measure emotions elicited by foods in pre-adolescents.

## 1. Introduction

Investigating children’s food preferences is important as they shape their eating behaviors, which can track into adulthood [1,2,3]. This requires age-appropriate and engaging tools [4], as children differ from adults in many aspects [5].

Consumer testing with children has mainly focused on hedonic measurements (acceptability of food products [4]). However, emotion measurements (how they feel about food products) have been shown to provide additional information beyond liking measurements that can help to better understand food experiences [6,7]. Only a small body of research has assessed food-elicited emotions with children ranging from 7–13 years [8,9,10,11,12,13]. These studies recognized the advantage of using emoji to measure food-elicited emotions, which are intuitive, easy-to-use and child-friendly. Emoji are supposed to simulate facial expressions that convey specific emotions or other situational meanings [14] that are language-independent indicators of emotions [15] and can help to elicit spontaneous expressions of emotional experiences elicited by foods [16].

Previous studies have selected emoji based on preliminary studies with children (e.g., using focus groups [10] or check-all-that-apply (CATA) [13]) or with adults (e.g., Schouteten et al. 2019). Although emoji were found to be able to discriminate between food products in children [9,12], little is known about how children interpret emoji in terms of their emotional meaning in the context of food.

An in-depth exploration of the meaning of emoji can contribute to understanding what is measured—if emotions or broader dimensions of product experience are measured, and if children agree in how they interpret the meaning of emoji. Research conducted with adults show a limited shared agreement in the meaning of emoji [17]. Intensive research by Jaeger, Ares and colleagues [18,19] on the meaning of 33 facial emoji found that there are emoji strongly associated with one emotion word, emoji that are associated with several but related emotions/moods and emoji that are associated with different moods and emotions, indicating multiple and unrelated meanings. Brants et al. [20] found a high agreement in the meaning for some emoji, such as *pouting face* (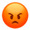), *crying face* 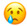, *face with open mouth* 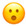, *neutral face* 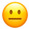 and *face with tears of joy* 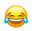 that were associated with a unique emotional meaning indicating respectively “anger,” “sadness,” “surprise,” “neutral” and “joy.” Interpretations of other emoji were found to depend on age or gender.

Research conducted with adults indicated that emoji differ in their affective meaning, spanning broad ranges in core valence and arousal [19,21]. Valence and arousal dimensions have been identified as two fundamental dimensions of meaning of core affect, and thus of emotions [22]. Valence refers to the differentiation between pleasant or unpleasant, while arousal refers to the level of physiological activation (high/low) [22]. Emotions are much more complex than valence and arousal, but emerge from the interaction of more basic mechanisms; valence and arousal are emergent properties of more basic processes [23]. Valence has been indicated as the *basic building block* that characterizes every emotion and that everybody is able to perceive [24], while the perception of arousal depends on the emotional granularity of the person experiencing an emotion. In fact, people differ in their ability to discriminate emotions based on activation. For example, individuals with low emotional granularity use more global terms when expressing their feelings and use the terms “sad” and “angry” interchangeably as a general expression of unpleasant feelings. In contrast, individuals with high emotional granularity make clearer distinctions between the terms that go beyond the description of an unpleasant feeling [24].

**Figure 1:**
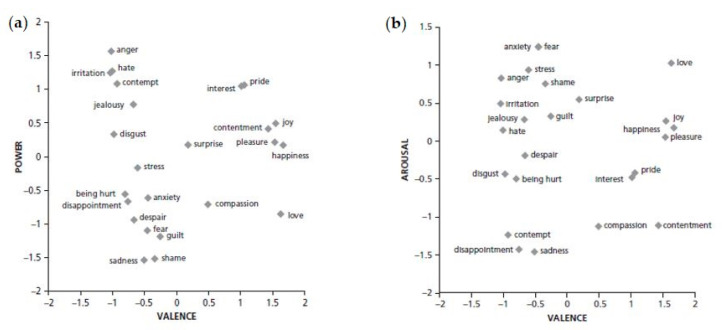
Comparison of plots displaying the valence vs. power dimension (**a**) and valence vs. arousal dimension (**b**) of 24 emotion words (source: Fontaine and Scherer, [25]).

Furthermore, research demonstrated the existence of a “power” dimension when studying the meaning of emotions [25]. The power dimension is defined by an opposition between control and lack of control of the situation, potency/strength vs. weakness and dominance vs. submissiveness: higher levels imply that the person has power over the situation and the people in the situation. For this reason, this dimension is different from arousal and can distinguish emotions characterized by similar arousal level, such as fear (low in power) and anger (high in power) that are both high in arousal (see Figure 1).

The ability to discriminate among emotions increases gradually with age, meaning that children’s emotion categories are quite broad and become more distinct during preschool years [26]. Age differences have been demonstrated in valence- and arousal ratings of emotion words in French children aged 7–13 years, where younger children (aged 7–9) gave higher mean valence- and arousal ratings compared to older children; moreover, the tendency to judge words positively (i.e., positive bias) decreased with age [27]. As we know that emotion vocabulary gradually becomes more stable and differentiated during middle childhood [28] and it is likely that some children are more limited in making fine distinctions between emotions, simplified questionnaires using emoji were suggested for evaluating emotions elicited by food products in this age group [29].

Furthermore, emotion expression can differ by gender in adults [30], but also in children [31]. Females have been shown to be more accurate than males in judging the emotional meaning of facial expressions [32] and to give higher valence and arousal ratings [33]. Gender differences were also found in studies investigating the interpretation of emoji [20] and in the selection of specific food-related emoji in pre-adolescents [13]. Women evaluated emoji as clearer and more meaningful and familiar [21] and gave higher overall positive ratings when assessing the valence of emoji [34]. They were also shown to be more familiar with emoji and to use them more frequently compared to men [21,34,35]. However, other studies did not report gender differences in the interpretation of emoji when using emoji questionnaires [36].

Hence, age- and gender differences in the meaning of emoji (including valence- and arousal dimensions) need to be further investigated as previous research shows controversial findings, leading to a further research question: Do gender and age influence how emoji are interpreted by pre-adolescents?

Semantic theories highlighted two perspectives on meaning: a differential dimension of meaning (intra-semiotic), defined by the inter-relationship of an object with other similar objects, and a cognitive dimension of meaning, which refers to the relationship with concepts [37]. This study aims at investigating the emotional meaning of food-related emoji in 9–13-year-old pre-adolescents. With the aim of covering both aspects of meaning we conducted two studies applying 1) a similarity-based method (projective mapping) to detect the meaning defined in the interrelationship between emoji (differential dimension meaning), and 2) a descriptive method (CATA) to investigate the association of emoji with words (cognitive/conceptual dimension of meaning).

In projective mapping, stimuli with different characteristics are mapped according to their similarities and differences. A set of stimuli is presented simultaneously and the respondent is asked to evaluate the stimulus and then position all stimuli on a two-dimensional space according to the similarities and differences between the stimuli [38]. This technique was widely used to describe food products [38,39] and could also be helpful when exploring similarities and differences of emoji because the respondent does not have to think about specific emotion words to describe each emoji. Additionally, it could give insights about their valence- and arousal dimensions or even other informative dimensions to uncover the meaning of emoji. Projective mapping has been previously used with children between 4 and 11 years to evaluate different food products and found to be appropriate for this age group because of its intuitive nature and suitability with untrained subjects [4,40,41]. This technique has never been applied before to determine similarities and differences of meanings of emoji in pre-adolescents or in adults.

The CATA approach was successfully used with adults to investigate the dominant meaning of emoji [18,19]. With pre-adolescents it has been used previously in connection with emoji to evaluate emotional experiences of food products [8,9,12], but not yet to understand their meaning.

Moreover, a further aim of the study was to measure age- and gender differences in the emotional meaning of food-related emoji.

## 2. Methods

The meaning of emoji was investigating using two different approaches, to cover two dimensions of meaning: differential (intra-semiotic) (study 1) and cognitive/conceptual (study 2).

### 2.1. Participants

A total of 254 children were recruited from three schools attending primary and secondary classes in the Florence area, Italy. 162 children participated in study 1 ((46% boys, aged 9–13 (*M* = 11, SD = 1.7, in both gender groups), whereas 92 participated in study 2 (57% boys) aged 11–13 (*M* = 12, SD = 0.3, in both gender groups).

Only children who returned a signed consent form from their parents and who agreed to voluntarily participate in the study by signing their own consent form were allowed to participate. There were no other exclusion criteria as no child should feel excluded from the study. Recruitment criteria and data treatment were planned in accordance to the General Data Protection Regulation (GDPR) 2016/679 and the principles of the Declaration of Helsinki. The protocol of the study was approved by the Ethical Committee of the University of Florence, Italy.

### 2.2. Data Collection

For both studies, testing took place in the regular classrooms of the schools. The tests lasted approximately one hour and were conducted during school time between 8AM and 3PM. The children sat in their normal seating order, either in rows or in groups of 2–6 seats. Children were given a tablet (Acer Iconia One 10, Android 7.0, Taiwan, China) and asked to fill in the questionnaire individually using the software Compusense Cloud (version 20.0.7557.33837, Compusense Inc., Guelph, ON, Canada).

During each testing session, one researcher led the test with the help of 2/3 assistants. The teacher was also present during the session to ensure the children were less distracted and felt at ease. Before each session, the researcher explained the questionnaire and made sure all children were confident about how to use the tablets. Children were invited to ask for assistance in case they had difficulties or questions.

### 2.3. Selection of Emoji

A preliminary study [13] was conducted to select emoji that were appropriate for 9–13-year-old children to describe their emotional experiences of foods. Table 1 displays the list of the selected 46 food-related emoji that were used in both studies (projective mapping and CATA).

### 2.4. Procedures

**Figure 2:**
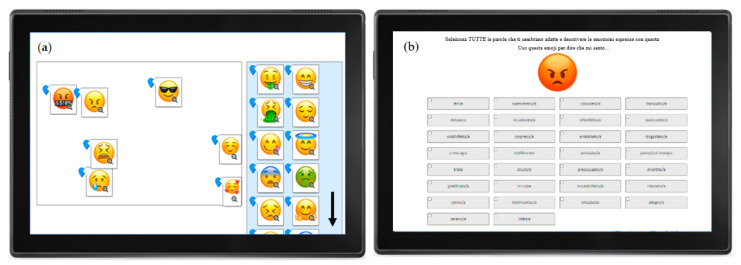
Examples (screenshots from the software Compusense Cloud) from Study 1—Projective Mapping of 46 emoji (**a**) and Study 2—CATA Questionnaire: 46 emoji described by emotion words (**b**).

The experimental setups of the projective mapping and CATA task are shown in Figure 2a,b. 

#### 2.4.1. Study 1—Projective Mapping

Children were asked to map the 46 emoji according to similarities and differences in their emotion meaning on a rectangular mapping area (dimensions: 140 × 90 mm) of the tablets. The instructions were: “These emoji were selected by other children as appropriate to describe food experiences. We are interested in knowing your opinion of how similar or different these emoji are in terms of meaning. Think about using these emoji to describe food experiences. Do not just think about the food you eat, because you also feel emotions for the foods you don′t eat (e.g., new foods that you have never tasted, hated foods etc.). You will see a white space on the screen on which to place the emoji. Arrange the emoji on the white space-the closer they are, the more similar their meaning is. It is also allowed to overlay emoji if they have a very similar meaning. The further away you place emoji, the more different their meaning is. Try to use all the space you have available. Pay attention to the meaning and not to how they look graphically. The exact position of each emoji is indicated by the blue pin. If you have any questions during the test, raise your hand and we will come to you.” Emoji were presented in a random order for each child. Children were able to freely choose in which order they wanted to map emoji and to rearrange emoji on the mapping area as much and as many times they wanted. An example was provided before the test with selected cartoons (PrEmo^®^ from Laurans and Desmet (2017) [43] expressing various emotions. The cartoons were printed in paper and the classroom’s blackboard was used by the lead researcher to explain to the children the basic concepts of a mapping task based on the same criteria of the study (similarities in meaning). The cartoons were used as an example instead of emoji to not introduce any bias in the study.

#### 2.4.2. Study 2—Check-All-That-Apply (CATA)

Children were asked to select all emotion words they found appropriate to express the meaning of each of the emoji. The 46 emoji were presented one at a time in two blocks of emoji (2 × 23 emoji). The presentation order of the two blocks was balanced across children. In between the two blocks children were given a refreshing break and they were asked to solve a riddle to decrease boredom effects. Emotion words were presented in a balanced order for each emoji. In case children could not find a fitting emotion word to describe the presented emoji or they felt that some words were missing, they had the possibility to specify their own emotion word in an open-ended response format. The instructions also emphasized that there were no right or wrong answers and that responses should be quick and spontaneous. Before the test started, the instructor went through the meaning of each emotion word for everyone in the classroom and poster with a child-friendly description of each emotion word was provided in the classroom. In case children expressed problems understanding a specific emotion word they could check the meaning on the poster and/or ask for questions to the researchers. These instructions appeared on the screen: “We are interested in the meaning of several emojis used to describe food experiences. You will be asked to evaluate 46 emojis in total but split into two sessions, so you will have a short break in between the two sessions. First, you will see on your screen an emoji and a list of 30 words. You will be asked to select all the words that seem suitable for you to describe the meaning of that emoji. You have to choose at least one word for each emoji, which best represents its meaning. You can also choose several words if you think they suit. If there is a word that is not included in the list but came to your mind, you can specify your own word on the next page. There are no right or wrong answers. If you have any questions during the test, raise your hand and we will come to you.”

##### Selection of Emotion Words

Emotion words were selected by reviewing literature on emotions elicited by foods [44,45] and general literature on emotions [46]. The selection intended to cover a wide range of emotion words differing in the valence- and arousal dimension (Table 2). Translation between languages was checked [47]. The final list resulted in 30 emotion words used in study 2.

#### 2.4.3. Emotion Usage Questionnaire (EUQ) and Test Evaluation

In both studies, children were asked about demographics (gender and age) and to fill in a questionnaire including emoji usage questions (EUQ) and questions about the test they completed [13]. Several domains of emoji usage were considered: Familiarity; Frequency of usage; Social use; Motivation; Valence; Enjoyment in using emoji. Two additional questions were asked to know how easy or difficult the test was (very difficult/a bit difficult/neither difficult nor easy/ easy/very easy) and how much they enjoyed it (by no means/a bit/so and so/fairly/a lot).

### 2.5. Data Analysis

Projective mapping was analyzed using a multi-configuration data analysis (STATIS) [49,50,51,52]. Age- and gender differences were analyzed by considering each subgroup (2 age groups: 9–11 years and 12–13 years; 2 gender groups: girls and boys) as a separate table.

To establish the meaning of emoji of the CATA questionnaire, Cochran’s Q test with Sheskin as post-hoc test was conducted. Frequency tables (emoji × CATA word combinations) were created and correspondence analysis (CA) was applied on the total sample and on boys and girls separated to evaluate gender differences (within-gender approach). RV coefficients were calculated to compare the plots by gender, age and methods. The interpretation of RV coefficients was accompanied by the visual inspection of the scatter plots of scores of the CAs conducted on each block of data, as recommended by Tomic et al. [53].

Hierarchical Multiple Factor Analysis (HMFA) was conducted to compare the results of projective mapping and of the CATA questionnaire (linking emoji to words).

Data from the EUQ were analyzed by computing frequencies (%) of responses. Gender and age effects on Familiarity, Frequency of usage, Valence and Enjoyment were tested by using Kruskal–Wallis One–Way Analysis of Variance by ranks. Chi-squared test was applied to test differences in the distributions of the responses between gender and age groups for each item of the other domains.

The level of significance for all the analysis was set at *p* ≤ 0.05. Statistical analyses were performed using XLSTAT (version 2018.7, Addinsoft, New York, NY, USA), except for HMFA that was conducted using RStudio (version 1.1.456, 2016, RStudio, Inc., Boston, MA, USA) and the package FactoMineR [54,55].

## 3. Results

### 3.1. Emoji Usage Questionnaire (EUQ)

Participants in both studies were found to be very familiar with emoji (71–77%, respectively, using them regularly) and used them either every day (56-66%) or a few times a week (29–27%), (Supplementary Materials Tables S1 and S2). Emoji were mostly used to communicate with friends (84–97%), parents (54–59%) and relatives (54–50%). The most important motivation to use emoji was because “they express something that normally cannot be described in words” (56–57%), “they make text messages more understandable” (51–62%) and “they are fun” (49–53%). The majority declared to use mostly positive emoji (78–55%) or both positive and negative (13–29%) in its communication and enjoys using emoji a lot (63–62%) or fairly enjoyed using them (32%).

Differences by gender were found in both studies. In study 1 a higher number of boys found that emoji “make text messages more understandable,” “save time when sending messages” and that “are quick to use” (domain Motivation). In study 2 gender differences were larger compared to study 1—especially in the domains Familiarity, Frequency of usage and Social use. Boys were more familiar (*p* = 0.003) with emoji in general and they had a higher Frequency of usage (*p* = 0.009). Furthermore, boys used emoji to communicate with parents (*p* = 0.005), siblings (*p* = 0.025) and relatives (*p* = 0.035) more than girls.

Significant age differences were detected in all domains (study 1), except in Valence. A higher number of older pre-adolescents used emoji more regularly (*p* = 0.001, Familiarity) and used them every day compared to younger pre-adolescents (*p* < 0.0001, Frequency of use). A higher frequency of older pre-adolescents sent emoji to friends (*p* < 0.0001) and parents (*p* = 0.033, Social use) and they also indicated that they use emoji because they “make the text messages more understandable” (*p* = 0.000) and “express something that normally can’t be described in words” (*p* = 0.006) more than younger pre-adolescents. In contrast, 68% of younger pre-adolescents compared to 33% of older pre-adolescents declared to use emoji because they are fun (*p* < 0.0001) (Motivation). This aligns with the domain Enjoyment, where 80% of younger pre-adolescents enjoyed using emoji compared to 50% of older pre-adolescents (*p* = 0.000).

### 3.2. Study 1—Projective Mapping

**Figure 3:**
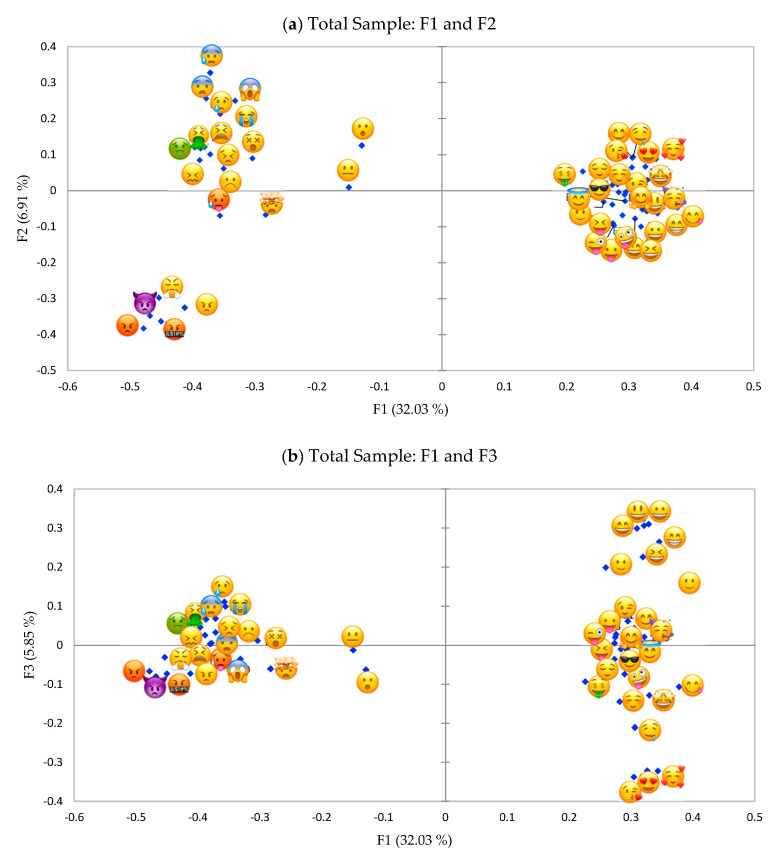
(**a**), Representation of emoji in the first and second dimension of the projective mapping task (study 1) of 46 food-related emoji evaluated by all pre-adolescents. (**b**), Representation of emoji in the first and third dimension of the projective mapping task (study 1) of 46 food-related emoji evaluated by all pre-adolescents.

Figure 3a shows results of the projective mapping task in which children mapped 46 food-related emoji according to their similarities and differences in their emotional meaning in the food context. The first two dimensions accounted for 38.95% of the variance. Visual inspection of the maps indicates that emoji were mainly separated on the first dimension which explained the 32.03% of the variance. Emoji were discriminated along the first dimension which can be interpreted as valence, with emoji commonly assumed having a positive meaning on the right of Figure 3a and emoji commonly expressing a negative meaning on the left side of Figure 3a. Emoji 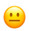 and 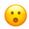, usually recognized as indicating “indifference” and “surprise” which are respectively low and high in arousal [19,20] but both neutral in the valence and power dimension [25], had an intermediate position in Figure 3a even if they were perceived as more similar to the negative emoji group (left side of Figure 3a).

Discrimination was also observed along the second dimension, which explained an additional 6.91% of the variance. However, the interpretation of the second dimension is less straightforward. Within emoji on the left side of the map of Figure 3a it is possible to identify on the bottom a group of emoji (
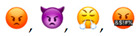
 and 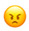) described in the literature as expressing “anger,” characterized by negative valence and high arousal [19,20]. The emotion “anger” is also high in the power dimension [25]. This group is opposed along the second dimension to a group of emoji (including 
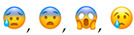
 and 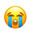), that were described in previous studies on adults [18,19,20] as indicating high to low arousal states and expressing different negative emotions which included “fear” and “sadness.” These emotions, while differing in arousal are both characterized by a low power [25].

Emoji on the right side of Figure 3a appear very close to each other, indicating a larger overlapping in terms of meaning compared to the group on the left side of Figure 3a. Within this group of emoji usually recognized as conveying a positive meaning it was possible to distinguish some emoji on the bottom right side of Figure 3a (e.g., 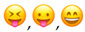 and 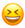 ) that previous studies on adults indicated as expressing “naughty/playful/goofy/mischievous” and “happy,” from other emoji on the top right side of Figure 3a (e.g., 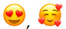) which were found to express “love” [18,19]. These two groups of emoji were all high in arousal, while they differed in power, being the former higher and the latter lower in power [25].

The third dimension explained an additional 5.85% of the variance (Figure 3b). This dimension differentiated mostly among the emoji conveying a positive meaning the ones commonly found expressing “happiness” (
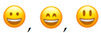
) and the ones expressing “love” (
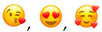
) based on previous study with adults [18,19], which are respectively characterized by lower and higher arousal. On the bottom left side of Figure 3b, in addition emoji indicating “anger” and “fear,” both higher in arousal but different in power, are now close to each other. These results taken together suggest that the second dimension can be interpreted as power, while the third dimension could be interpreted as arousal.

### 3.3. Gender- and Age Differences

**Figure 4:**
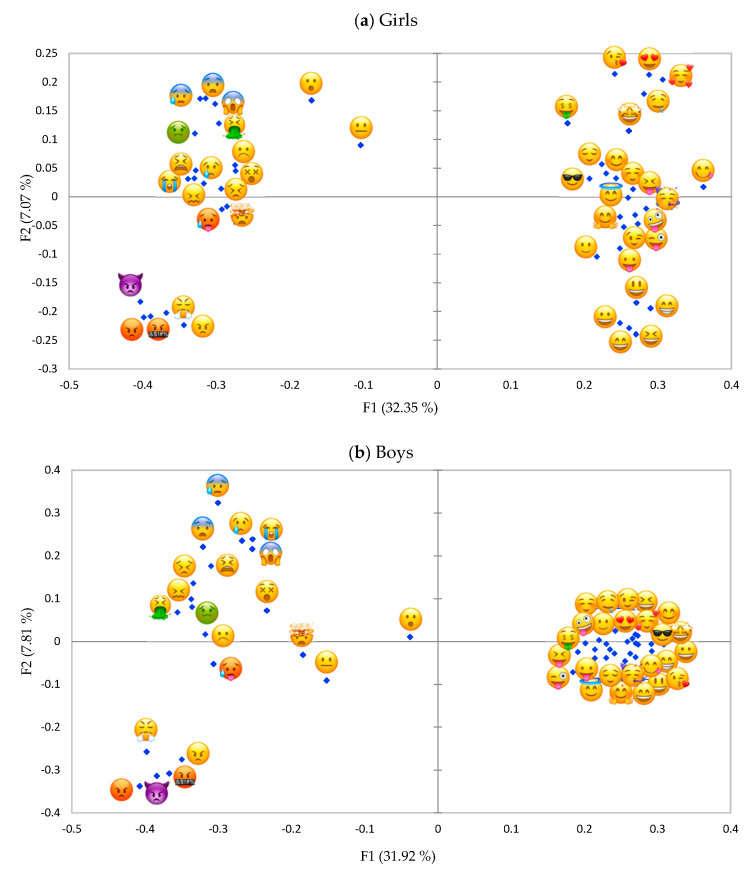
(**a**), Representation of emoji in the first and second dimension of the projective mapping task (study 1) of 46 food-related emoji evaluated by girls (*n* = 87). (**b**) Representation of emoji in the first and second dimension of the projective mapping task (study 1) of 46 food-related emoji evaluated by boys (*n* = 75).

**Figure 5:**
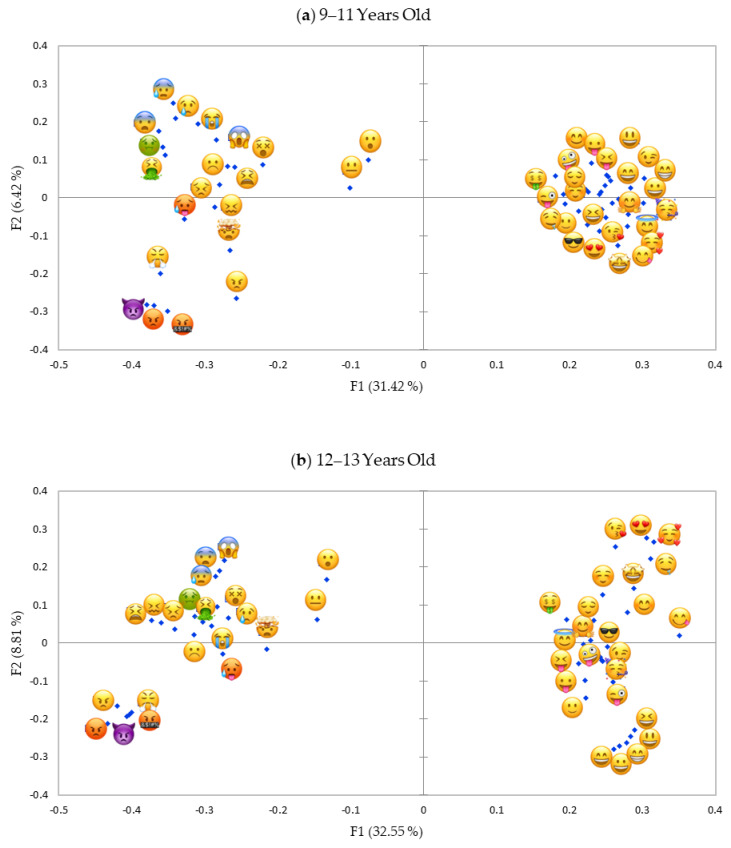
(**a**), Representation of emoji in the first and second dimension of the projective mapping task (study 1) of 46 food-related emoji evaluated by 9–11-year-old pre-adolescents (*n* = 75). (**b**), Representation of emoji in the first and second dimension of the projective mapping task (study 1) of 46 food-related emoji evaluated by 12–13-year-old pre-adolescents (*n* = 87).

Configuration of emoji in segments divided by age- and gender (Figure 4a,b and Figure 5a,b) were similar to Figure 3a (all children). Explained variance of the first two dimensions ranged between 37.84% and 41.36% considering children separated by gender (boys and girls) and age group (9–11 y.o. and 12–13 y.o.), with the first dimension explaining 31.42–32.55% of the variance. The maps were compared by gender and age calculating the RV coefficient and through visual inspection. RV coefficients were very high in both cases (0.94 for gender and 0.92 for age). Visual inspection of the map indicated that gender- and age differences were mainly observed on the second dimension, that we interpreted as power. Girls and older pre-adolescents (12–13 y.o.) were able to discriminate between positive emoji much better showing some emoji indicating “love” [18] that were low in power (e.g., 
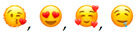
) and some emoji indicating “happy” and thus that were higher in power (e.g., 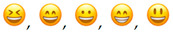). For boys and younger pre-adolescents (9–11 y.o.) this discrimination among positive emoji could not be observed. The emoji *face with open mouth* 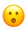 was interpreted as quite neutral by boys, while it was perceived as more similar to negative emoji by girls. On the other hand, the emoji *neutral face* 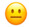 had a more negative meaning for boys as demonstrated by the fact that the emoji was closer (= more similar) to the negative emoji in boys than in girls.

### 3.4. Study 2—CATA with Emoji

Each emoji was described by 11.73 words on average, with 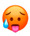 and 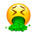 that were described by the lowest number of words (5.47 and 5.51, respectively) and the emoji 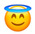 and 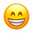 that were described by the highest number of words (17.20 and 17.83, respectively). Furthermore, on average the number of emotion words selected was higher for positive emoji than for negative emoji (14.29 and 8.67, respectively).

All emotion words discriminated significantly among emoji (*p* < 0.0001, Supplementary Materials Table S3). The results confirmed that the separation on the first dimension in the figure of projective mapping (Figure 3a,b) can be interpreted as valence. The interpretation of the meaning of the negative emoji was more straightforward, than the one of positive emotions.

The “angry” emoji group was shown to have by far the most shared emotional meaning (
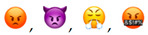
 and 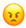), where 74–95% of pre-adolescents agreed on the emotion word “angry,” and for the last three also on the emotion word “annoyed.” The *nauseated face* 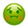 (87% of respondents) and the *face vomiting* 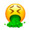 (84% of respondents) were mostly associated with “disgusted.” The *face vomiting* 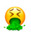 was also described by its physical appearances such as “vomiting” and by a “sick” feeling (12% respondents of open-ended response, respectively).

A group of negative emoji (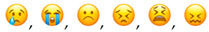) was shown to have multiple meaning. All express “unhappy” (49–66%) and “disappointed” (39–47%) while the first two indicated also with large agreement “sad” (75% and 86%, respectively). The last two expressed also “guilty” (40% and 47% respectively). The *face screaming fear* 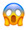 was mainly described by “surprised” (58%) and “worried” (48%) but an additional 22% of pre-adolescents described the emoji as “scared/frightened” in the open comments (“fear” was not included in the emotion list). The *fearful face* 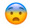 was described as “worried” (56%) and “surprised” (42%).

The *neutral face* 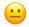 was mainly associated with “indifferent” (58% of respondents) and the *face with open mouth* 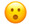 with “surprised” (73% of respondents). The *dizzy face* 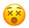 was associated both with surprised (40%) and with worried (40%), suggesting a more negative meaning.

The emoji 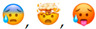 and 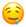 were not clearly associated with any emotion word (each emotion word was checked by less than 40% of the participants). All other emoji were associated with “happy” (33-78%) and many of them also with “cheerful” with large agreement. The emoji 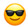 expressed in addition “confident” and “at ease” (50 and 44%, respectively). The emoji 
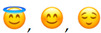
 were also associated with “calm” (44–47%) and “serene” (47–60%). The emoji 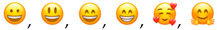 were associated with “serene” (44–55%) and “cheerful” (42–71%), while the emoji 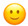 and 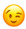 were associated mostly with serene (49 and 36%, respectively). The emoji 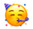 and 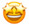 expressed a combination of “happy” (70 and 64%), “cheerful” (70 and 49%), “enthusiastic” (49 and 48%), “energetic” (52 and 54%), “amused” (49–42%). The emoji 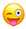, 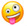, 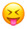 expressed a combination of “happy” (59–63%), “amused” (51–53%), “cheerful” (57–59%) and “energetic“ (41–57%). Emoji 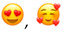 and 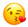 were used also to express “in love” based on the further comments provided by the participants (63%, 35% and 17%, respectively).

**Figure 6:**
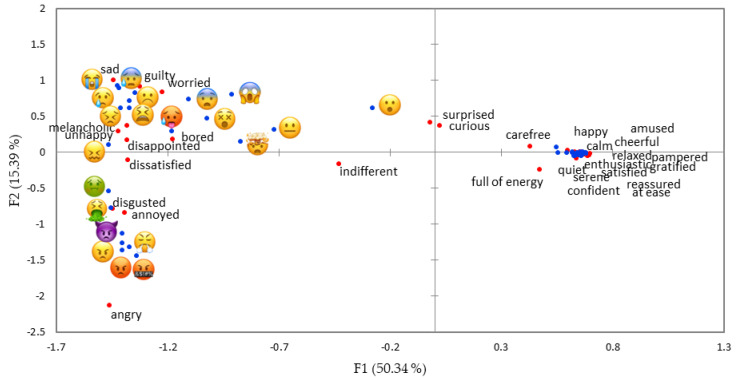
Emoji and words representation in the first and second dimensions of the correspondence analysis of CATA task (study 2), where 46 food-related emoji were described by emotion words by 12–13-year-old pre-adolescents. Emoji 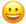, 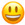, 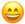, 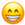, 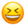, 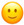, 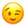, 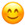, 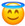, 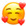, 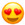, 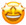, 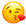, 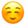, 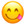, 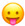, 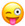, 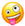, 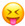, 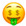, 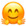, 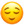, 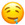, 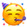 and 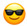 are not shown on the right side of the figure because they are overlapped.

The configuration of emoji based on CATA with words (Figure 6) was very similar to the one of study 1 using projective mapping (Figure 3a), but the explained variance of the two first dimensions was higher. The first two dimensions accounted for 65.74% of variance. Emoji were mainly divided along the first dimension (F1: 50.34%) between positive and negative (valence), but negative emoji were also discriminated along the second dimension (F2: 15.39%). Similar to the projective mapping task the second dimension seemed to be described more by power than arousal and discriminated mainly among the negative emoji (on the left side of Figure 6). Positive emoji appeared as overlapped on the map. No major differences by gender were found neither by inspecting the maps nor through RV coefficient (=0.93).

### 3.5. Comparing Study 1 and Study 2: Hierarchical Multiple Factor Analysis

**Figure 7:**
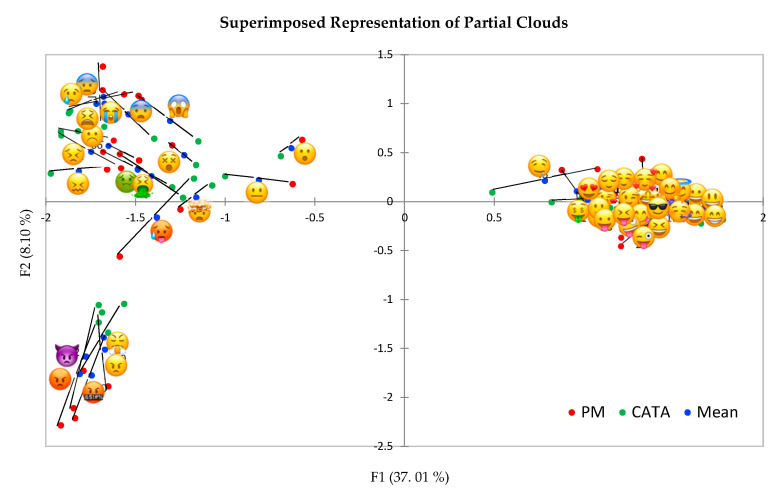
Superimposed representation of the partial clouds resulting from Hierarchical Multiple Factor Analysis (HMFA) comparing projective mapping (PM) (study 1) and CATA (study 2).

The superimposed representation of the partial clouds shows that the two tasks gave quite similar results (Figure 7), as demonstrated also by the RV coefficient = 0.95. Small differences were observed on the first dimension for the emoji 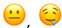, and some other emoji on the top left of Figure 7, and on the second dimension particularly for some negative emoji (the “angry” group and 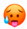). Overall, projective mapping discriminated slightly better than CATA the “angry” group, 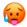 and 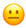, while in other cases in which a difference was found, CATA contributed to further discrimination.

## 4. Discussion

The current study aimed at investigating the emotional meaning of emoji used to describe food experiences in 9–13-year-old pre-adolescents. Familiarity and frequency of use of emoji was very high (over 70% use them regularly), particularly for boys and older pre-adolescents (12–13 y.o.), confirming that emoji are a common communication tool for this age range.

Taken together, the results showed that the selected emoji were mainly discriminated according to valence (positive vs. negative). However, they were also differentiated according to the power dimension (control vs. lack of control of the situation) and, to a lower extent, according the arousal dimension (high vs. low activation). In general, negative emoji were better discriminated in their emotional meaning than positive emoji. This could be explained by the fact that the span of perceptual differences of the emoji expressing positive meaning was smaller than the one of the emoji with a negative meaning. We may also hypothesize that children found it easier to discriminate between negative emotions than between positive emotions, due to their emotional development. In fact, children acquire emotion categories gradually, but it has not been demonstrated if negative or positive emotions are acquired first [26]. However, most of what we know about the development of emotion categories comes from studies of infant attention to highly stereotypical, posed facial configurations, ignoring the real-world variability [56]. Furthermore, most of the studies on emotional development in children are limited to basic emotions, thus including only one positive emotion (happiness). On the other hand, these differences in discrimination ability between positive and negative emoji were not found in adults [18,19], thus indicating a specificity of this age range. Further studies are needed to better explain this finding.

Both methods provided consistent results and contributed to the understanding of the meaning of emoji in pre-adolescents. Projective mapping provided information on the semantic inter-relationships between emoji according to the dimensions structuring emotion meaning, while CATA allowed to investigate the association between emoji and words. Furthermore, from a methodological point of view, projective mapping was found to be very effective in determining similarities and differences of meanings of emoji in pre-adolescents and could be recommended as a method to explore the meaning of emoji.

### 4.1. Meaning of Emoji: Similarities and Differences

Emoji were primarily separated according to valence on the first component, which is in agreement with other research that identified the valence dimension in emoji [19,21,25,57]. Valence is the building block of emotional life [24] and this may explain this result. Emoji conveying a positive meaning were clearly separated from emoji conveying a negative meaning indicating that emoji have a distinct meaning in terms of valence, and this was true regardless of gender and age. With the aim of linking results from projective mapping and CATA, in this section we will refer to the emotions associated with the emoji based on results of study 2.

The categorization in positive and negative emoji was consistent with previous studies [10]. Additionally, neutral or moderately valanced emoji were found [19,21] including the two emoji indicating “indifferent” and “surprised”. For their positioning on the map, these emoji resulted as the most neutral compared to the others, which conforms with previous studies [19,21], even if they were interpreted as slightly more negative (more similar to the negative emoji than to the positive emoji). Surprise can be both positive and negative [25,58], while indifference in the case of food experience is generally associated with a not optimal product performance [59]. Emoji faces with neutral facial expressions are often used in questionnaires/scales with the intent to have neither a positive nor negative meaning [11,60], but attention has to be paid when used with pre-adolescents as they might interpret these emoji as more negative than expected.

Differently from previous studies on adults [18], the second dimension cannot be interpreted as arousal. This dimension can instead be interpreted as power for negative emoji and to some minor extent for positive emoji. In fact, the second dimension separated emoji expressing “angry” and “annoyed” from emoji expressing “sad” and “worried”/“surprised”/“fear”. Power, and not arousal, discriminate anger from fear: both are high in arousal, but the former is high in power as well, while the latter is low in power. Sadness is the opposite to fear in terms of arousal but they both are low in power and this may explain their close positioning on the map. The emoji expressing “disgusted,” characterized by an intermediate level of power, were situated between the two groups described above. Positive emoji were not discriminated very well along the second dimension. However, some emoji on the bottom right side (Figure 3a) expressing “cheerful”/“amused”/“happy”/“energetic” tended to be higher in power, whereas other emoji on the top right side (Figure 3a) expressing “love” tended to be lower in power. The third dimension differentiated better the emoji conveying a positive meaning and might indicate arousal, discriminating the ones expressing “happy”/“serene”, low in arousal, from the ones expressing “happy”/“love,” higher in arousal. This could be observed to a lower extent also on the left side of Figure 3b, where we can see that emoji expressing “angry” and “worried”/“surprised”/“fear” are now close to each other (both are high in arousal) and separated from emoji indicating “sad” (low arousal).

This difference in the relevance of the dimension of power in relation to the dimension of arousal may be explained by a different focus on aspects of the meaning of emoji in pre-adolescents compared to adults. In this age range the attention could be more on the control of the situation than on the physiological activation. This finding is also consistent with the theory of emotions as constructions made by a person to give meaning to the sensations he or she experiences [24].

### 4.2. Associations with Words

The CATA study provided similar results to study 1, but this study allowed to link words to the emoji to better investigate their meaning. Compared to adults, the frequency of selection of the emotion words was considerably higher [18]. This may be due to age-related characteristics and may also be related to the context in which the task was performed—at school, in which usually children are required to pay attention and be concentrated.

Negative emoji resulted in clearer and distinct emotional meanings compared to positive emoji, but also positive emoji were differentiated showing different nuances in meaning. The word “happy” was selected for all emoji conveying a positive meaning. Pre-adolescents may use “happy” to describe general feelings of pleasantness, which is suitable to characterize most positive emoji. This word might be in fact the most familiar for children to indicate a positive emotion and was shown previously to be very accurately identified in emoji by 4–8-year-old children without experience in social media or smartphone use [61].

In many cases more than one emoji could be used to express similar meanings with relatively high agreement between participants. It was also shown that some emoji express a meaning that is not limited to an emotion word. This is for example the case of some emoji that expressed both “angry” and “annoyed”. This is in line with Jaeger et al. [18] that showed the existence of multiple meanings for several emoji and associations between emoji and emotion words differing in strength. As noted, many positive emoji differ slightly graphically and expressed probably different intensities of emotions compared to negative emoji that in many cases indicated different discrete emotions. This is the case for example of the emoji 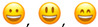 and 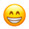 that expressed all “happy”, “cheerful” and “serene” (frequency > than 40%), with small differences, even if not statistically significant: the percentage of association of “cheerful” with the last two emoji was 71 and 64%, respectively.

In many cases we observed similar meanings compared to previous studies [18,20], indicating an overall agreement in the use between adults and pre-adolescents. However, in some cases we found additional meanings: for example, the emoji 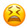 and 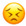 were associated not only with “unhappy”, “sad” and “disappointed” but also with “guilty.” Furthermore, the emoji 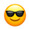 was used to express to be “happy”, “at ease” and “confident.”

Our results indicated also that a limited number of emoji should be excluded from studies with pre-adolescents as their meaning is unclear and not shared among this age group. Some emoji could be less familiar in general, but it should also be considered that pre-adolescents may differ in their experience in how they use emoji [13] and this may impact how their meaning is interpreted; e.g., there may be differences in social interaction or social media platforms leading to various “learned” meanings of the same emoji. This might explain in part the heterogeneous responses for the same emoji.

The results indicate that, especially in the case of positive valence, emoji that are used in questionnaires to measure emotional responses to food products should be selected with caution in order to avoid possible risks of misinterpretation or interpretation discrepancies.

### 4.3. Gender and Age Differences

Results showed that differences in gender and age were limited. All pre-adolescents independently from gender and age agreed on the discrimination on the first component, distinguishing emoji expressing a positive from emoji expressing a negative emotion. In the projective mapping task girls and older pre-adolescents (12–13 y.o.) discriminated according to the power dimension better among positive emoji compared to boys and younger pre-adolescents (9–11 y.o).

This could be related to gender differences in children’s emotional expression [31]. Girls may be higher in emotional granularity of positive emotions and therefore being able to make finer distinctions between positive emoji. Girls express more positive emotions than boys which further develop with increasing age [31]. Previous research showed that pre-adolescent girls selected several emoji more frequently when asked about how they feel about foods consumed in different eating contexts [13]. Girls tended to use more positive emoji (83%) compared to boys (72%), but no significant differences were found.

The slight improvement in discrimination ability reported with age could be due to an improved cognitive development [62] and ability to distinguish between emotion categories. This suggests that older pre-adolescents are higher in emotional granularity for positive emoji, while they are both granular for negative emoji. Moreover, older pre-adolescents were shown to be higher in user experience, familiarity and social use of emoji. By using emoji more frequently, children may learn what they mean in different contexts thereby improving their ability to make finer distinctions between emoji meanings (increased emotional granularity). Most older children (12–13 y.o.) declared that emoji make their text messages more understandable and that they express something that normally cannot be described in words; a similar trend was reported by Sick et al. [13].

Altogether, these findings suggest that the meaning of food-related emoji is clearer to older (12–13 y.o.) than younger pre-adolescents (9–11 y.o.) as they were found to be more able to discriminate not only among emoji expressing a negative meaning but also among emoji expressing a positive meaning. Hence, self-report questionnaires using emoji to measure responses to foods should be used with caution in younger pre-adolescents (9–11 y.o.) as the meaning of emoji (especially positive emoji) is less clear to them with specific attention to avoid ambiguity and overlapping between emoji. However, as we did not include younger pre-adolescents in study 2, further research is recommended to test the ability of 9–11-year-old children to link emoji with emotion words.

## 5. Conclusions

The study contributed with new insights to uncover the meaning of food-related emoji evaluated by 9–13-year-old pre-adolescents. Our findings indicated that pre-adolescents discriminated emoji to express food experiences according to valence (between positive vs. negative) and, to a lower extent, to power (control vs. lack of control) and arousal dimension (activation vs. absence of activation). In general, negative emoji were better discriminated in their emotional meaning than positive emoji. Specific emotion words were associated to negative emoji, whereas for positive emoji this association was less clear. Some positive emoji express a mix of emotions. In the projective mapping task, girls and older pre-adolescents (12–13 y.o.) discriminated positive emoji slightly better along the power dimension compared to boys and younger pre-adolescents (9–11 y.o.), which could be related to an increased emotional granularity and user experience.

These findings can be used to develop a self-report questionnaire for pre-adolescents to measure emotions in response to food experiences. Identifying similarities and differences among emoji can help to detect clusters of emoji expressing similar emotional meanings to avoid redundancy and thus to reduce the length of emoji lists used in such questionnaires. Having a clearer picture of the meaning of emoji for this age range may help to limit sources of ambiguities to improve the effectiveness of this approach. Furthermore, considering a broad range of emoji differing in distinct emotional meanings and dimensions (including valence, power and arousal) can result in meaningful emotional profiles necessary to discriminate between food products when used with pre-adolescents.

Taken together, these results suggest that there are emoji whose meaning is less clear than others and that should not be included in questionnaires. Many emoji have similar or partially overlapping meanings, while others have a very specific meaning. This information could be used to select emoji for a self-report questionnaire to be used with pre-adolescents to measure their emotional experiences of food products.

## Figures and Tables

**Table 1:** Emoji list of 46 emoji appropriate to describe food experiences in pre-adolescents resulting from Sick et al. [13] with emoji names from Emojipedia [42].

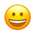	*grinning face*	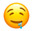	*drooling face*
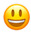	*grinning face with big eyes*	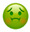	*nauseated face*
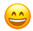	*grinning face with smiling eyes*	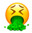	*face vomiting*
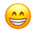	*beaming face with smiling eyes*	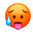	*hot face*
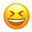	*grinning squinting face*	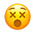	*dizzy face*
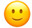	*slightly smiling face*	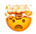	*exploding face*
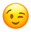	*winking face*	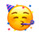	*partying face*
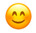	*smiling face with smiling eyes*	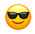	*smiling face with sunglasses*
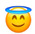	*smiling face with halo*	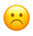	*frowning face*
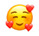	*smiling face with hearts*	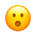	*face with open mouth*
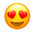	*smiling face with heart-eyes*	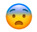	*fearful face*
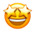	*star-struck*	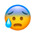	*anxious face with sweat*
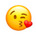	*face blowing a kiss*	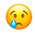	*crying face*
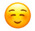	*smiling face*	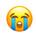	*loudly crying face*
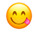	*face savoring food*	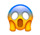	*face screaming fear*
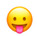	*face with tongue*	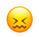	*confounded face*
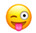	*winking face with tongue*	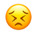	*persevering face*
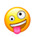	*zany face*	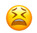	*tired face*
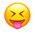	*squinting face with tongue*	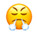	*face with steam from nose*
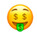	*money-mouth face*	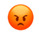	*pouting face*
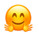	*hugging face*	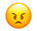	*angry face*
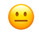	*neutral face*	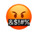	*face with symbols on mouth*
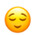	*relieved face*	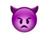	*angry face with horns*

**Table 2:** Selection of emotion words following the 12-point circumplex structure of core affect.

Dimensions	Emotion Words		
	References	Study 2 (English)	Study 2 (Italian)
I Pleasant Activation	energetic ^1,2,3^, excited ^1,2^, sensual ^3^	energetic	pieno di energia
II Activated Pleasure	enthusiastic ^1,2^, elated^1^, inspired ^2^, amused ^3^, cheerful ^3^	enthusiastic, amused, cheerful	entusiasta, allegro/a, divertito/a
III Pleasure	pleased ^1^, satisfied ^1,2,3^, happy ^2,3^, happy memory ^3^, merry, cuddled ^3^, gratified ^3^	happy, satisfied, cuddled, gratified	felice, soddisfatto/a, coccolato/a, gratificato/a
IV Deactivated Pleasure	serene ^1^, peaceful ^1^, secure ^2,3^, at ease ^2^, generous ^3^, tender ^3^, anti-stress ^3^ (calming, shooting, reassuring)	confident, at ease, reassured	sicuro/a, a mio agio, rassicurato/a
V Pleasant Deactivation	placid ^1^, tranquil ^1^, relaxed ^2,3^/carefree ^3^, calm ^2^	relaxed, calm, serene, carefree	rilassato/a, calmo/a, sereno/a, spensierato/a
VI Deactivation	quiet ^1,2^, still ^1^, passive ^2^, indifferent ^3^	indifferent, quiet	indifferente, tranquillo/a
VII Unpleasant Deactivation	sluggish ^1^,tired ^1^, dulled ^2^, bored ^2,3^	bored	annoiato/a
VIII Deactivated Displeasure	sad ^1,3^, gloomy ^1^, blue ^2^, uninspired ^2^	sad, melancholic	triste, malinconico/a
IX Displeasure	unhappy ^1,2^, dissatisfied ^1,2^, neglected ^3^, disappointed ^3^	unhappy, dissatisfied, disappointed	infelice, insoddifatto/a, deluso/a
X Activated Displeasure	distressed ^1^, upset ^1^, tense ^2^, bothered ^2^, guilty ^3^	guilty	in colpa
XI Unpleasant Activation	frenzied ^1^, jittery ^1, 2^, nervous ^2^, annoyed ^3^	annoyed, disgusted, angry, worried	infastidito/a, disgustato/a, arrabbiato/a, preoccupato/a
XII Activation	aroused ^1^, activated ^1^, active ^2^, alert ^2^, surprised ^3^, curious ^3^	surprised, curious	sorpreso/a, incuriosito/a

^1^ [46]; ^2^ [48]; ^3^ [44].

## Supplementary Materials

The following are available online at https://www.mdpi.com/2304-8158/9/9/1307/s1, Table S1: Emoji usage questionnaire of study 1 (projective mapping): Familiarity; Frequency of usage; Social use; Motivation; Valence of emoji; Enjoyment in using emoji. Total frequencies (%) and frequencies divided by genders (girls and boys) and age (9–11 and 12–13). Depending on data analysis *p* values refer to Kruskal Wallis (*) or Chi-square test. In bold *p* values < 0.05, Table S2: Emoji usage questionnaire of study 2 (CATA questionnaire): Familiarity; Frequency of usage; Social use; Motivation; Valence of emoji; Enjoyment in using emoji. Total frequencies (%) and frequencies divided by genders (girls and boys). Depending on data analysis *p* values refer to Kruskal Wallis (*) or Chi-square test. In bold *p* values < 0.05, Table S3: Frequency use (%) for each of the 46 facial emojis by each of the 30 CATA terms. In the last raw additional words provided by children are reported. Frequencies >20% are marked in bold. Different letters (in brackets) indicate a significant difference according to Sheskin post-hoc test (*p* < 0.05). Additional words (grouped by their meaning) were mentioned by >10% of children in the open-ended response option.

## References

[1] Ventura A.K., Worobey J. (2013). Early Influences on the Development of Food Preferences. Curr. Boil..

[2] de Cosmi V., Scaglioni S., Agostoni C. (2017). Early Taste Experiences and Later Food Choices. Nutrients.

[3] Harris G. (2008). Development of taste and food preferences in children. Curr. Opin. Clin. Nutr. Metab. Care.

[4] Laureati M., Pagliarini E., Toschi T.G., Monteleone E. (2015). Research challenges and methods to study food preferences in school-aged children: A review of the last 15years. Food Qual. Prefer..

[5] Mortimer J.T., Shanahan M.J. (2003). Handbook of the Life Course.

[6] King S.C., Meiselman H.L. (2010). Development of a method to measure consumer emotions associated with foods. Food Qual. Prefer..

[7] Spinelli S., Monteleone E. (2018). Emotional Responses to Products. Methods in Consumer Research.

[8] Schouteten J.J., Verwaeren J., Lagast S., Gellynck X., de Steur H. (2018). Emoji as a tool for measuring children’s emotions when tasting food. Food Qual. Prefer..

[9] Gallo K.E., Swaney-Stueve M., Chambers E. (2017). Comparing visual food images versus actual food when measuring emotional response of children. J. Sens. Stud..

[10] Gallo K.E., Swaney-Stueve M., Chambers E. (2017). A focus group approach to understanding food-related emotions with children using words and emojis. J. Sens. Stud..

[11] Swaney-Stueve M., Jepsen T., Deubler G. (2018). The emoji scale: A facial scale for the 21st century. Food Qual. Prefer..

[12] Schouteten J.J., Verwaeren J., Gellynck X., Almli V.L. (2019). Comparing a standardized to a product-specific emoji list for evaluating food products by children. Food Qual. Prefer..

[13] Sick J., Spinelli S., Dinnella C., Monteleone E. (2020). Children’s selection of emojis to express food-elicited emotions in varied eating contexts. Food Qual. Prefer..

[14] Bai Q., Dan Q., Mu Z., Yang M. (2019). A Systematic Review of Emoji: Current Research and Future Perspectives. Front. Psychol..

[15] Novak P.K., Smailović J., Sluban B., Mozetič I., Kralj Novak P., Smailović J., Sluban B., Mozetič I. (2015). Sentiment of emojis. PLoS ONE.

[16] Vidal L., Ares G., Jaeger S.R. (2016). Use of emoticon and emoji in tweets for food-related emotional expression. Food Qual. Prefer..

[17] Franco C.L., Fugate J.M.B. (2020). Emoji Face Renderings: Exploring the Role Emoji Platform Differences have on Emotional Interpretation. J. Nonverbal. Behav..

[18] Jaeger S.R., Ares G. (2017). Dominant meanings of facial emoji: Insights from Chinese consumers and comparison with meanings from internet resources. Food Qual. Prefer..

[19] Jaeger S.R., Roigard C.M., Jin D., Vidal L., Ares G. (2018). Valence, arousal and sentiment meanings of 33 facial emoji: Insights for the use of emoji in consumer research. Food Res. Int..

[20] Brants W., Sharif B., Serebrenik A. (2019). Assessing the Meaning of Emojis for Emotional Awareness-A Pilot Study. Companion Proceedings of the 2019 World Wide Web Conference.

[21] Rodrigues D., Prada M., Carvalho R., Garrido M.V., Lopes D. (2017). Lisbon Emoji and Emoticon Database (LEED): Norms for emoji and emoticons in seven evaluative dimensions. Behav. Res. Methods.

[22] Russell J.A., Barrett L.F. (1999). Core affect, prototypical emotional episodes, and other things called emotion: Dissecting the elephant. J. Pers. Soc. Psychol..

[23] Barrett L.F. (2016). Navigating the Science of Emotion. Emotion Measurement.

[24] Barrett L.F. (2006). Valence is a basic building block of emotional life. J. Res. Pers..

[25] Fontaine J.R.J., Scherer K.R. (2013). The global meaning structure of the emotion domain: Investigating the complementarity of multiple perspectives on meaning1. Components of Emotional Meaning.

[26] Widen S.C., Russell J.A. (2008). Children acquire emotion categories gradually. Cogn. Dev..

[27] Monnier C., Syssau A. (2016). Affective norms for 720 French words rated by children and adolescents (FANchild). Behav. Res. Methods.

[28] Bahn D., Vesker M., Alanis J.C.G., Schwarzer G., Kauschke C. (2017). Age-Dependent Positivity-Bias in Children’s Processing of Emotion Terms. Front. Psychol..

[29] Spinelli S., Jaeger S.R. (2019). What do we know about the sensory drivers of emotions in foods and beverages?. Curr. Opin. Food Sci..

[30] Kring A.M., Gordon A.H. (1998). Sex Differences in Emotion: Expression, Experience, and Physiology. J. Pers. Soc. Psychol..

[31] Chaplin T.M., Aldao A. (2013). Gender differences in emotion expression in children: A meta-analytic review. Psychol. Bull..

[32] Hall J.A., Matsumoto D. (2004). Gender Differences in Judgments of Multiple Emotions from Facial Expressions. Emotion.

[33] Proverbio A.M. (2016). Sex differences in social cognition: The case of face processing. J. Neurosci. Res..

[34] Jones L.L., Wurm L.H., Norville G.A., Mullins K.L. (2020). Sex differences in emoji use, familiarity, and valence. Comput. Hum. Behav..

[35] Chen Z., Lu X., Ai W., Li H., Mei Q., Liu X. (2018). Through a Gender Lens. Proceedings of the 2018 World Wide Web Conference on World Wide Web-WWW’18.

[36] Jaeger S.R., Xia Y., Lee P.Y., Hunter D.C., Beresford M.K., Ares G. (2018). Emoji questionnaires can be used with a range of population segments: Findings relating to age, gender and frequency of emoji/emoticon use. Food Qual. Prefer..

[37] Violi P. (2001). Meaning and Experience.

[38] Valentin D., Chollet S., Lelièvre M., Abdi H. (2012). Quick and dirty but still pretty good: A review of new descriptive methods in food science. Int. J. Food Sci. Technol..

[39] Varela P., Ares G. (2012). Sensory profiling, the blurred line between sensory and consumer science. A review of novel methods for product characterization. Food Res. Int..

[40] Varela P., Salvador A. (2014). Structured sorting using pictures as a way to study nutritional and hedonic perception in children. Food Qual. Prefer..

[41] Mitterer-Daltoé M.L., Breda L.S., Belusso A.C., Nogueira B.A., Rodrigues D.P., Fiszman S., Varela P. (2017). Projective mapping with food stickers: A good tool for better understanding perception of fish in children of different ages. Food Qual. Prefer..

[42] Emojipedia Apple Emoji List. https://emojipedia.org/apple/.

[43] Laurans G., Desmet P.M.A. (2017). Developing 14 animated characters for non-verbal self-report of categorical emotions. J. Des. Res..

[44] Spinelli S., Masi C., Dinnella C., Zoboli G.P., Monteleone E. (2014). How does it make you feel? A new approach to measuring emotions in food product experience. Food Qual. Prefer..

[45] Jaeger S.R., Lee P.-Y., Xia Y., Chheang S.L., Roigard C.M., Ares G. (2019). Using the emotion circumplex to uncover sensory drivers of emotional associations to products: Six case studies. Food Qual. Prefer..

[46] Yik M., Russell J.A., Steiger J.H. (2011). A 12-point circumplex structure of core affect. Emotion.

[47] Scherer K.R., Wallbott H.G., Summerfield A.B., Scherer K.R. (1986). Experiencing Emotion: A Cross-Cultural Study.

[48] Jaeger S.R., Spinelli S., Ares G., Monteleone E. (2018). Linking product-elicited emotional associations and sensory perceptions through a circumplex model based on valence and arousal: Five consumer studies. Food Res. Int..

[49] Abdi H., Williams L.J., Valentin D., Bennani-Dosse M. (2012). STATIS and DISTATIS: Optimum multitable principal component analysis and three way metric multidimensional scaling. Wiley Interdiscip. Rev. Comput. Stat..

[50] McHale D., Lavit C. (1990). Analyse Conjointe de Tableaux Quantitatifs. Biometrics.

[51] Lavit C., Escoufier Y., Sabatier R., Traissac P. (1994). The ACT (STATIS method). Comput. Stat. Data Anal..

[52] Nestrud M.A., Lawless H.T. (2010). Perceptual Mapping of apples and cheeses using projective mapping and sorting. J. Sens. Stud..

[53] Tomic O., Berget I., Næs T. (2015). A comparison of generalised procrustes analysis and multiple factor analysis for projective mapping data. Food Qual. Prefer..

[54] Lé S., Josse J., Husson F. (2008). FactoMineR: An R Package for Multivariate Analysis. J. Stat. Softw..

[55] R Core Team (2016). R: A Language and Environment for Statistical Computing.

[56] Hoemann K., Wu R., LoBue V., Oakes L.M., Xu F., Barrett L.F. (2020). Developing an Understanding of Emotion Categories: Lessons from Objects. Trends Cogn. Sci..

[57] Jaeger S.R., Roigard C.M., Ares G. (2018). Measuring consumers’ product associations with emoji and emotion word questionnaires: Case studies with tasted foods and written stimuli. Food Res. Int..

[58] Kim H., Somerville L.H., Johnstone T., Polis S., Alexander A.L., Shin L.M., Whalen P.J. (2004). Contextual Modulation of Amygdala Responsivity to Surprised Faces. J. Cogn. Neurosci..

[59] Spinelli S., Masi C., Zoboli G., Prescott J., Monteleone E. (2015). Emotional responses to branded and unbranded foods. Food Qual. Prefer..

[60] Laureati M., Bergamaschi V., Pagliarini E. (2015). Assessing childhood food neophobia: Validation of a scale in Italian primary school children. Food Qual. Prefer..

[61] Oleszkiewicz A., Frackowiak T., Sorokowska A., Sorokowski P. (2017). Children can accurately recognize facial emotions from emoticons. Comput. Hum. Behav..

[62] Eccles J.S. (1999). The development of children ages 6 to 14. Future Child..

