# Supplementary material for: The Meaning of Emoji to Describe Food Experiences in Pre-Adolescents
Source: Foods. 2020 Sep 16;9(9):1307. doi: 10.3390/foods9091307 (PMC7555141; doi:10.3390/foods9091307)
Supplement: Supplementary file 1 [file foods-09-01307-s001.pdf]

**Table S1.** Emoji usage questionnaire of Study 1 (projective mapping): Familiarity; Frequency of usage; Social use; Motivation; Valence of emoji; Enjoyment in using emoji. Total frequencies (%) and frequencies divided by genders (girls and boys) and age (9–11 and 12–13). Depending on data analysis *p* values refer to Kruskal Wallis (*) or Chi-square test. In bold *p* values ≤ 0.05.

| Question | | Domain | Scale Categories/Items | Frequency (%) | | | | | | |
|---|---|---|---|---|---|---|---|---|---|---|
| | | | | | Gender | | | Age | | |
| | | | | **Total (*n* = 162)** | **Girls (*n* = 87)** | **Boys (*n* = 75)** | ***p* Value** | **9-11 (*n* = 75)** | **12-13 (*n* = 87)** | ***p* Value** |
| 1 | Did you know what emoji were before participating in this study? | Familiarity | I use them regularly | 71 | 68 | 76 | 0.271* | 60 | 82 | **0.001*** |
| | | | I use them occasionally | 23 | 27 | 18 | | 30 | 17 | |
| | | | I had used them but only once | 2 | 1 | 3 | | 4 | 0 | |
| | | | I had heard of them but had never used them | 1 | 1 | 0 | | 1 | 0 | |
| | | | I never heard of them | 3 | 3 | 3 | | 5 | 1 | |
| 2 | How often do you usually use emoji? | Frequency of usage | every day | 56 | 57 | 55 | 0.963* | 32 | 77 | **<0.0001*** |
| | | | a few times a week | 29 | 24 | 33 | | 40 | 18 | |
| | | | a couple of times a month or less | 10 | 15 | 5 | | 19 | 4 | |
| | | | never | 5 | 4 | 7 | | 9 | 1 | |
| 3 | To whom do you send emoji? | Social use | friends | 84 | 83 | 85 | 0.656 | 68 | 98 | **<0.0001** |
| | | | siblings | 35 | 31 | 39 | 0.308 | 28 | 40 | 0.103 |
| | | | parents | 54 | 54 | 55 | 0.935 | 45 | 62 | **0.033** |
| | | | relatives | 54 | 55 | 52 | 0.686 | 48 | 59 | 0.176 |
| | | | teachers | 2 | 5 | 0 | 0.060 | 3 | 2 | 0.880 |
| | | | other | 22 | 21 | 24 | 0.613 | 27 | 18 | 0.206 |
| 4 | Why do you use emoji? | Motivation | they are fun | 49 | 45 | 55 | 0.212 | 68 | 33 | **<0.0001** |
| | | | I can use emoji instead of words | 41 | 38 | 44 | 0.433 | 39 | 43 | 0.618 |
| | | | they make my text messages more understandable | 51 | 43 | 61 | **0.017** | 36 | 64 | **0.000** |
| | | | to save time when sending messages | 23 | 14 | 33 | **0.003** | 21 | 24 | 0.672 |
| | | | they highlight a part of the message | 30 | 31 | 28 | 0.673 | 24 | 34 | 0.145 |
| | | | they express something I normally can't describe in words, e.g. how I feel | 56 | 56 | 55 | 0.833 | 44 | 66 | **0.006** |
| | | | they are quick to use | 25 | 17 | 33 | **0.018** | 25 | 24 | 0.860 |
| 5 | Do you use more emoji with positive or negative meaning? | Valence | positive | 78 | 83 | 72 | 0.131* | 78 | 78 | 0.850* |
| | | | negative | 1 | 1 | 1 | | 1 | 1 | |
| | | | almost the same | 13 | 8 | 19 | | 12 | 14 | |
| | | | I don't know | 8 | 8 | 8 | | 9 | 7 | |
| 6 | How much do you like using emoji? | Enjoyment | a lot | 63 | 67 | 60 | 0.573* | 80 | 50 | **0.000*** |
| | | | fairly | 32 | 26 | 39 | | 19 | 44 | |
| | | | so and so | 4 | 6 | 1 | | 3 | 5 | |
| | | | a bit | 1 | 1 | 0 | | 0 | 1 | |
| | | | by no means | 0 | 0 | 0 | | 0 | 0 | |

**Table S2.** Emoji usage questionnaire of Study 2 (CATA questionnaire): Familiarity; Frequency of usage; Social use; Motivation; Valence of emoji; Enjoyment in using emoji. Total frequencies (%) and frequencies divided by genders (girls and boys). Depending on data analysis $p$ values refer to Kruskal Wallis (*) or Chi-square test. In bold $p$ values $\leq$ 0.05.

| | Question | Domain | Scale Categories/Items | Frequency | | | |
|---|---|---|---|---|---|---|---|
| | | | | **Total ($n$ = 92)** | **Girls ($n$ = 52)** | **Boys ($n$ = 40)** | ***p*-Value** |
| **1** | Did you know what emoji were before participating in this study? | Familiarity | I use them regularly | 77 | 65 | 92 | **0.003*** |
| | | | I use them occasionally | 20 | 31 | 5 | |
| | | | I had used them but only once | 0 | 0 | 0 | |
| | | | I had heard of them but had never used them | 1 | 0 | 3 | |
| | | | I never heard of them | 2 | 4 | 0 | |
| **2** | How often do you usually use emoji? | Frequency of usage | every day | 66 | 56 | 80 | **0.009*** |
| | | | a few times a week | 27 | 32 | 20 | |
| | | | a couple of times a month or less | 6 | 10 | 0 | |
| | | | never | 1 | 2 | 0 | |
| **3** | To whom do you send emoji? | Social use | friends | 97 | 94 | 100 | 0.122 |
| | | | siblings | 47 | 37 | 60 | **0.025** |
| | | | parents | 59 | 46 | 75 | **0.005** |
| | | | relatives | 50 | 40 | 63 | 0.035 |
| | | | teachers | 1 | 0 | 3 | 0.252 |
| | | | other | 26 | 27 | 25 | 0.835 |
| **4** | Why do you use emoji? | Motivation | they are fun | 53 | 60 | 45 | 0.164 |
| | | | I can use emoji instead of words | 39 | 33 | 48 | 0.149 |
| | | | they make my text messages more understandable | 62 | 60 | 65 | 0.598 |
| | | | to save time when sending messages | 15 | 13 | 18 | 0.593 |
| | | | they highlight a part of the message | 28 | 29 | 28 | 0.887 |
| | | | they express something I normally can't describe in words, e.g. how I feel | 57 | 56 | 58 | 0.868 |
| | | | they are quick to use | 22 | 29 | 13 | 0.060 |
| **5** | Do you use more emoji with positive or negative meaning? | Valence | positive | 55 | 62 | 45 | 0.131 |
| | | | negative | 1 | 2 | 0 | |
| | | | almost the same | 29 | 23 | 38 | |
| | | | I don't know | 15 | 13 | 17 | |
| **6** | How much do you like using emoji? | Enjoyment | a lot | 62 | 56 | 70 | 0.123* |
| | | | fairly | 32 | 34 | 28 | |
| | | | so and so | 4 | 6 | 2 | |
| | | | a bit | 1 | 2 | 0 | |
| | | | by no means | 1 | 2 | 0 | |

**Table S3.** Frequency of use (%) for each of the 46 facial emojis by each of the 30 CATA terms. In the last raw additional words provided by children are reported. Frequencies >20% are marked in bold. Different letters (in brackets) indicate a significant difference according to Sheskin post-hoc test ($p < 0.05$). Additional words (grouped by their meaning) were mentioned by >10% of children in the open-ended response option.

| | | | | | | | | | | | | | | | | | | | | | | | |
|---|---|---|---|---|---|---|---|---|---|---|---|---|---|---|---|---|---|---|---|---|---|---|---|
| Curious | 8 (ab) | 8 (ab) | 4 (ab) | 5 (ab) | 5 (ab) | 3 (ab) | 4 (ab) | 5 (ab) | 5 (ab) | 4 (ab) | 4 (ab) | 5 (ab) | 1 (a) | 3 (ab) | 7 (ab) | 7 (ab) | 5 (ab) | 7 (ab) | 3 (ab) | 3 (ab) | 5 (ab) | 2 (ab) | 14 (b) |
| Surprised | 28 (defghi) | 32 (efghi) | 21 (bcdefgh) | 19 (abcdef) | 15 (abcde) | 4 (abc) | 9 (abcd) | 10 (abcd) | 8 (abc) | 13 (abcde) | 15 (abcde) | 37 (fghi) | 8 (abc) | 4 (abc) | 15 (abcde) | 11 (abcd) | 11 (abcd) | 23(cdefghi) | 20 (abcdefg) | 15 (abcde) | 17 (abcdef) | 3 (abc) | 13 (abcde) |
| Energetic | 25 (cdefg) | 35 (efgh) | 26 (defg) | 37 (fghi) | 38 (fghi) | 4 (abc) | 11 (abcd) | 8 (abcd) | 11 (abcd) | 7 (abcd) | 14 (abcde) | **54 (hi)** | 10 (abcd) | 1 (a) | 24 (bcdefg) | 25 (cdefg) | **41 (ghi)** | **57 (i)** | **41 (ghi)** | 39 (fghi) | 15 (abcde) | 7 (abcd) | 5 (abcd) |
| Enthusiastc | 33 (efghi) | 40 (ghi) | 25 (cdefgh) | **42 (hi)** | 40 (ghi) | 9 (abcd) | 20 (abcdefg) | 21 (abcdefg) | 25 (cdefgh) | 21 (abcdefg) | 34 (efghi) | **48 (i)** | 20 (abcdefg) | 13(abcde) | 22 (bcdefgh) | 29 (defghi) | 32 (efghi) | 36 (fghi) | 37 (fghi) | 26 (cdefgh) | 33 (efghi) | 7 (abc) | 16 (abcdef) |
| Cheerful | **50 (efghi)** | **55 (fghi)** | **71 (i)** | **64 (hi)** | **64 (hi)** | 35 (cdefg) | 40 (cdefgh) | **52 (fghi)** | **44 (cdefgh)** | **42 (cdefgh)** | 32 (cdef) | **49 (defghi)** | 36 (cdefg) | 26 (bcde) | **51 (efghi)** | **42 (cdefgh)** | **59 (ghi)** | **58 (ghi)** | **57 (fghi)** | **42 (cdefgh)** | **48 (defghi)** | 21 (abc) | 24 (abcd) |
| Amused | **41 (hijklm)** | 37 (ghijkl) | **45 (ijklm)** | **60 (m)** | **47 (jklm)** | 20 (abcdefgh) | 32 (efghijkl) | 20 (abcdefgh) | 23 (bcdefghi) | 10 (abcde) | 10 (abcde) | **42 (ijklm)** | 19 (abcdefg) | 15 (abcdefg) | 24 (cdefghi) | **53 (lm)** | **51 (klm)** | **53 (lm)** | **52 (klm)** | 28 (defghij) | 33 (fghijkl) | 11 (abcdef) | 8 (abcd) |
| Happy | **66 (efg)** | **78 (g)** | **75 (fg)** | **70 (efg)** | **66 (efg)** | **61 (efg)** | **49 (cdef)** | **64 (efg)** | **55 (cdefg)** | **61 (efg)** | **59 (defg)** | **64 (efg)** | **46 (cde)** | **46 (cde)** | **59 (defg)** | **58 (defg)** | **59 (defg)** | **60 (defg)** | **63 (efg)** | **51 (cdefg)** | **54 (cdefg)** | 33 (cd) | 29 (bc) |
| Satisfied | 22 (cdef) | 24 (defg) | 19 (abcdef) | 27 (defgh) | 14 (abcde) | 22 (cdef) | 27 (defgh) | 24 (defg) | 36 (fgh) | 20 (abcdef) | 16 (abcdef) | 36 (fgh) | 15 (abcde) | 21 (bcdef) | 27 (defgh) | 15 (abcde) | 21 (bcdef) | 9 (abcd) | 19 (abcdef) | **46 (h)** | 26 (defgh) | 29 (efgh) | 26 (defgh) |
| Cuddled | 10 (ab) | 9 (ab) | 7 (ab) | 9 (ab) | 3(ab) | 4 (ab) | 4 (ab) | 27 (def) | 13 (abcd) | **58 (g)** | 33 (ef) | 7 (ab) | 35 (f) | 39 (f) | 8 (ab) | 1 (a) | 1 (a) | 2 (a) | 7 (ab) | 2 (a) | 26 (cdef) | 19 (bcde) | 14 (abcd) |
| Gratified | 17 (cdef) | 20 (def) | 17 (cdef) | 20 (def) | 7 (abcd) | 11 (abcde) | 10 (abcde) | 24 (ef) | 30 (f) | 15 (abcdef) | 12 (abcde) | 30 (f) | 16 (bcdef) | 17 (cdef) | 10 (abcde) | 1 (ab) | 7 (abcd) | 5 (abcd) | 7 (abcd) | 20 (def) | 20 (def) | 15 (abcdef) | 13 (abcde) |
| Confident | 32 (fghi) | 27 (efgh) | 28 (efgh) | 34 (ghi) | 19 (abcdefg) | 17 (abcdefg) | 40 (hi) | 24 (defgh) | 32 (fghi) | 20 (bcdefg) | 14 (abcdef) | 20 (bcdefg) | 21 (cdefg) | 26 (efgh) | 11 (abcde) | 16 (abcdefg) | 32 (fghi) | 16 (abcdefg) | 20 (bcdefg) | 20 (bcdefg) | 20 (bcdefg) | 35 (ghi) | 5 (abcd) |
| At Ease | 26 (cde) | 35 (cde) | 34 (cde) | 23 (bcde) | 17 (abcd) | 35 (cde) | 35 (cde) | 37 (de) | 37 (de) | 33 (cde) | 15 (abc) | 24 (bcde) | 34 (cde) | 34 (cde) | 25 (cde) | 23 (bcde) | 29 (cde) | 25 (cde) | 24 (bcde) | 20 (abcd) | 35 (cde) | 36 (cde) | 20 (abcd) |
| Reassured | 13 (abcdefgh) | 16 (bcdefgh) | 23 (fgh) | 21 (efgh) | 4 (abcd) | 9 (abcdef) | 16 (bcdefgh) | 28 (h) | 16 (bcdefgh) | 20 (defgh) | 7 (abcde) | 10 (abcdef) | 16 (bcdefgh) | 26 (gh) | 12 (abcdefg) | 7 (abcde) | 9 (abcdef) | 5 (abcde) | 8 (abcdef) | 1 (abcdefgh) | 19 (cdefgh) | 23 (fgh) | 5 (abcde) |
| Carefree | 16 (abcdefg) | 15 (abcdefg) | 19 (abcdefg) | 17 (abcdefg) | 19 (abcdefg) | 17 (abcdefg) | 20 (bcdefg) | 14 (abcdefg) | 26 (fg) | 27 (fg) | 16 (abcdefg) | 15 (abcdefg) | 12 (abcdefg) | 17 (abcdefg) | 16 (abcdefg) | 14 (abcdefg) | 24 (defg) | 27 (fg) | 19 (abcdefg) | 16 (abcdefg) | 16 (abcdefg) | 22 (cdefg) | 30 (g) |
| Relaxed | 24 (defghijk) | 23 (cdefghijk) | 28 (fghijk) | 23 (cdefghijk) | 7 (abcd) | 29 (ghijk) | 21 (bcdefghijk) | 19 (abcdefghij) | 39 (k) | 30 (hijk) | 11 (abcdefg) | 8 (abcde) | 21 (bcdefghijk) | 32 (ijk) | 13 (abcdefghi) | 9 (abcde) | 10 (abcdef) | 7 (abcd) | 12 (abcdefgh) | 16 (abcdefghij) | 26 (efghijk) | 39 (k) | 39 (jk) |
| Calm | 30 (efgh) | 29 (efgh) | 27 (defgh) | 26 (cdefg) | 11 (abcde) | 39 (fgh) | 29 (efgh) | **44 (gh)** | **47 (h)** | 38 (fgh) | 12 (abcde) | 4 (ab) | 29 (efgh) | 40 (fgh) | 15 (abcde) | 16 (abcde) | 12 (abcde) | 5 (ab) | 8 (abcd) | 5 (ab) | 30 (efgh) | **44 (gh)** | 22 (bcdef) |
| Serene | **54 (hij)** | **44 (efghij)** | **55 (ij)** | **45 (efghij)** | 32 (defgh) | **49 (efghij)** | **36 (defghi)** | **47 (efghij)** | **60 (j)** | **44 (efghij)** | 33 (defghi) | 26 (bcde) | 38 (defghij) | 39 (defghij) | 33 (defghi) | 36 (defghi) | 38 (defghi) | 19 (abcd) | 29 (defg) | 32 (defgh) | **50 (fghij)** | **52 (ghij)** | 27 (cdef) |
| Indifferent | 7 (ab) | 3 (a) | 4 (a) | 1 (a) | 7 (ab) | 19 (b) | 1 (a) | 2 (a) | 2 (a) | 3 (a) | 0 (a) | 0 (a) | 5 (ab) | 3 (a) | 3 (a) | 4 (a) | 1 (a) | 3 (a) | 3 (a) | 4 (a) | 4 (a) | 8 (ab) | 3 (a) |
| Quiet | 27 (efghij) | 21 (bcdefghij) | 23 (cdefghij) | 29 (fghij) | 11 (abcdef) | 35 (ijk) | 22 (cdefghij) | 32 (ghijk) | 50 (k) | 26 (defghij) | 15 (abcdefgh) | 10 (abcde) | 26 (defghij) | 32 (ghijk) | 16 (abcdefghi) | 15 (abcdefgh) | 21 (bcdefghij) | 10(abcde) | 13 (abcdefg) | 10(abcde) | 30(ghij) | 39 (jk) | 24 (defghij) |
| Bored | 2 (ab) | 0 (a) | 0 (a) | 0 (a) | 1 (a) | 1 (a) | 0 (a) | 0 (a) | 0 (a) | 1 (a) | 0 (a) | 0 (a) | 1 (a) | 1 (a) | 1 (a) | 0 (a) | 0 (a) | 0 (a) | 0 (a) | 0 (a) | 1 (a) | 2 (ab) | 4 (ab) |
| Sad | 2 (a) | 0 (a) | 0 (a) | 1 (a) | 0 (a) | 0 (a) | 0 (a) | 0 (a) | 0 (a) | 1 (a) | 0 (a) | 0 (a) | 0 (a) | 0 (a) | 0 (a) | 0 (a) | 1 (a) | 0 (a) | 0 (a) | 1 (a) | 0 (a) | 0 (a) | 2 (a) |
| Melancholic | 2 (ab) | 0 (a) | 1 (a) | 2 (ab) | 0 (a) | 1 (a) | 0 (a) | 0 (a) | 0 (a) | 1 (a) | 0 (a) | 1 (a) | 0 (a) | 0 (a) | 0 (a) | 0 (a) | 0 (a) | 0 (a) | 0 (a) | 0 (a) | 1 (a) | 0 (a) | 2 (ab) |
| Unhappy | 2 (ab) | 0 (a) | 1 (ab) | 0 (a) | 0 (a) | 1 (ab) | 0 (a) | 1 (ab) | 0 (a) | 1 (ab) | 0 (a) | 1 (ab) | 0 (a) | 1 (ab) | 1 (ab) | 1 (ab) | 0 (a) | 0 (a) | 0 (a) | 2 (ab) | 0 (a) | 0 (a) | 0 (a) |
| Dissatisfied | 1 (a) | 1 (a) | 0 (a) | 1 (a) | 1 (a) | 1 (a) | 0 (a) | 0 (a) | 0 (a) | 1 (a) | 0 (a) | 0 (a) | 0 (a) | 0 (a) | 1 (a) | 1 (a) | 1 (a) | 0 (a) | 1 (a) | 0 (a) | 0 (a) | 0 (a) | 2 (a) |
| Disappointed | 1 (a) | 1 (a) | 1 (a) | 1 (a) | 0 (a) | 1 (a) | 0 (a) | 0 (a) | 1 (a) | 2 (a) | 0 (a) | 0 (a) | 0 (a) | 1 (a) | 0 (a) | 0 (a) | 1 (a) | 0 (a) | 0 (a) | 1 (a) | 0 (a) | 1 (a) | 2 (a) |
| Annoyed | 1 (a) | 0 (a) | 0 (a) | 1 (a) | 1 (a) | 2 (ab) | 0 (a) | 0 (a) | 0 (a) | 1 (a) | 0 (a) | 0 (a) | 0 (a) | 1 (a) | 1 (a) | 1 (a) | 0 (a) | 0 (a) | 1 (a) | 0 (a) | 0 (a) | 0 (a) | 0 (a) |
| Disgusted | 3 (ab) | 0 (a) | 0 (a) | 0 (a) | 0 (a) | 0 (a) | 0 (a) | 1 (ab) | 0 (a) | 1 (ab) | 0 (a) | 0 (a) | 0 (a) | 1 (ab) | 1 (ab) | 0 (a) | 0 (a) | 0 (a) | 3 (ab) | 0 (a) | 0 (a) | 0 (a) | 1 (ab) |
| Angry | 3 (ab) | 0 (a) | 1 (a) | 1 (a) | 1 (a) | 1 (a) | 0 (a) | 0 (a) | 0 (a) | 1 (a) | 0 (a) | 0 (a) | 0 (a) | 0 (a) | 0 (a) | 0 (a) | 0 (a) | 0 (a) | 0 (a) | 0 (a) | 0 (a) | 0 (a) | 0 (a) |
| Worried | 3 (abc) | 0 (a) | 0 (a) | 1 (ab) | 1 (ab) | 3 (abc) | 0 (a) | 0 (a) | 0 (a) | 2 (ab) | 0 (a) | 0 (a) | 0 (a) | 0 (a) | 0 (a) | 0 (a) | 0 (a) | 0 (a) | 0 (a) | 0 (a) | 1 (ab) | 0 (a) | 0 (a) |
| Guilty | 2 (a) | 0 (a) | 1 (a) | 2 (a) | 0 (a) | 0 (a) | 1 (a) | 1 (a) | 1 (a) | 1 (a) | 1 (a) | 0 (a) | 0 (a) | 1 (a) | 2 (a) | 0 (a) | 0 (a) | 0 (a) | 0 (a) | 1 (a) | 1 (a) | 1 (a) | 0 (a) |
| Additional Words | - | - | - | - | - | - | - | - | I am good / I feel like an angel (11%) | in love (35%) | in love (63%) | - | in love (17%) | - | gluttonous (14%), hungry (10%) | - | - | - | - | rich (26%) | - | - | - |

*Cont.*

| | | | | | | | | | | | | | | | | | | | | | | | |
|---|---|---|---|---|---|---|---|---|---|---|---|---|---|---|---|---|---|---|---|---|---|---|---|
| Curious | 1 (a) | 5 (ab) | 4 (ab) | 1 (a) | 2 (ab) | 0 (a) | 7 (ab) | 3 (ab) | 1 (a) | 35 (c) | 3 (ab) | 5 (ab) | 5 (ab) | 1 (a) | 7 (ab) | 0 (a) | 1 (a) | 9 (ab) | 0 (a) | 0 (a) | 1 (a) | 2 (ab) | 2 (ab) |
| Surprised | 23 (cdefghi) | 7 (abc) | 5 (abc) | 1 (ab) | 2 (ab) | 4 (abc) | 40 (hij) | 39 (ghij) | 0 (a) | **73 (k)** | **42 (ij)** | 8 (abc) | 2 (ab) | 0 (a) | **58 (jk)** | 2 (ab) | 4 (abc) | 7 (abc) | 2 (ab) | 1 (ab) | 2 (ab) | 3 (abc) | 1 (ab) |
| Energetic | **52 (hi)** | 26 (defg) | 0 (a) | 0 (a) | 0 (a) | 4 (ab) | 1 (a) | 10 (abcd) | 0 (a) | 2 (a) | 1 (a) | 0 (a) | 0 (a) | 0 (a) | 2 (a) | 1 (a) | 0 (a) | 0 (a) | 4 (abc) | 14 (abcde) | 9 (abcd) | 19 (abcdef) | 10 (abcd) |
| Enthusiastic | **49 (i)** | 20 (abcdefg) | 0 (a) | 0 (a) | 1 (ab) | 1 (ab) | 3 (ab) | 7 (abc) | 0 (a) | 5 (abc) | 0 (a) | 1 (ab) | 0 (a) | 0 (a) | 3 (ab) | 0 (a) | 2 (ab) | 0 (a) | 1 (ab) | 0 (a) | 0 (a) | 0 (a) | 0 (a) |
| Cheerful | **70 (i)** | 35 (cdefg) | 0 (a) | 0 (a) | 0 (a) | 0 (a) | 0 (a) | 1 (ab) | 0 (a) | 1 (ab) | 0 (a) | 0 (a) | 0 (a) | 1 (ab) | 2 (ab) | 0 (a) | 0 (a) | 0 (a) | 0 (a) | 0 (a) | 0 (a) | 0 (a) | 0 (a) |
| Amused | **49 (jklm)** | 30 (efghijk) | 0 (a) | 0 (a) | 0 (a) | 0 (a) | 0 (a) | 1 (ab) | 0 (a) | 3 (abc) | 0 (a) | 0 (a) | 0 (a) | 1 (ab) | 0 (a) | 0 (a) | 0 (a) | 0 (a) | 1 (ab) | 0 (a) | 0 (a) | 1 (ab) | 0 (a) |
| Happy | **70 (efg)** | **49 (cdef)** | 0 (a) | 0 (a) | 0 (a) | 0 (a) | 1 (a) | 0 (a) | 0 (a) | 3 (ab) | 0 (a) | 0 (a) | 0 (a) | 2 (ab) | 1 (a) | 0 (a) | 0 (a) | 0 (a) | 0 (a) | 0 (a) | 0 (a) | 0 (a) | 0 (a) |
| Satisfied | 26 (defgh) | 44 (gh) | 0 (a) | 1 (ab) | 0 (a) | 0 (a) | 1 (ab) | 1 (ab) | 1 (ab) | 3 (abc) | 0 (a) | 0 (a) | 1 (ab) | 0 (a) | 1 (ab) | 1 (ab) | 1 (ab) | 0 (a) | 0 (a) | 0 (a) | 0 (a) | 0 (a) | 1 (ab) |
| Cuddled | 11 (abc) | 3 (ab) | 0 (a) | 1 (a) | 0 (a) | 0 (a) | 0 (a) | 0 (a) | 0 (a) | 1 (a) | 0 (a) | 0 (a) | 0 (a) | 0 (a) | 0 (a) | 0 (a) | 0 (a) | 0 (a) | 0 (a) | 0 (a) | 0 (a) | 0 (a) | 0 (a) |
| Gratified | 16 (bcdef) | 12 (abcde) | 3 (abc) | 0 (a) | 0 (a) | 2 (abc) | 1 (ab) | 0 (a) | 0 (a) | 1 (ab) | 1 (ab) | 0 (a) | 0 (a) | 2 (abc) | 0 (a) | 0 (a) | 0 (a) | 1 (ab) | 0 (a) | 0 (a) | 1 (ab) | 1 (ab) | 0 (a) |
| Confident | 17 (abcdefg) | **50 (i)** | 2 (abc) | 0 (a) | 1 (ab) | 1 (ab) | 0 (a) | 0 (a) | 0 (a) | 3 (abc) | 1 (ab) | 0 (a) | 0 (a) | 0 (a) | 0 (a) | 0 (a) | 0 (a) | 0 (a) | 3 (abc) | 3 (abc) | 3 (abc) | 2 (abc) | 1 (ab) |
| At Ease | 26 (cde) | **44 (e)** | 1 (a) | 0 (a) | 0 (a) | 1 (a) | 1 (a) | 0 (a) | 0 (a) | 3 (ab) | 0 (a) | 0 (a) | 0 (a) | 1 (a) | 0 (a) | 0 (a) | 1 (a) | 0 (a) | 1 (a) | 1 (a) | 1 (a) | 0 (a) | 1 (a) |
| Reassured | 10 (abcdef) | 11 (abcdefg) | 3 (abc) | 0 (a) | 0 (a) | 0 (a) | 0 (a) | 1 (ab) | 1 (ab) | 3 (abc) | 1 (ab) | 1 (ab) | 2 (ab) | 0 (a) | 0 (a) | 0 (a) | 0 (a) | 1 (ab) | 1 (ab) | 0 (a) | 0 (a) | 0 (a) | 1 (ab) |
| Carefree | 25 (efg) | 24 (defg) | 12 (abcdefg) | 2 (ab) | 0 (a) | 2 (ab) | 2 (ab) | 7 (abcde) | 5 (abcd) | 4 (abc) | 9 (abcdef) | 9 (abcdef) | 2 (ab) | 4 (abc) | 3 (abc) | 3 (abc) | 2 (ab) | 7 (abcde) | 2 (ab) | 0 (a) | 1 (ab) | 2 (ab) | 0 (a) |
| Relaxed | 13 (abcdefghi) | 34 (jk) | 5 (abcd) | 0 (a) | 0 (a) | 2 (ab) | 0 (a) | 1 (a) | 2 (ab) | 4 (abc) | 0 (a) | 0 (a) | 1 (a) | 0 (a) | 0 (a) | 0 (a) | 0 (a) | 0 (a) | 0 (a) | 0 (a) | 0 (a) | 0 (a) | 0 (a) |
| Calm | 7 (abc) | 27 (defgh) | 15 (abcde) | 0 (a) | 1 (a) | 4 (ab) | 1 (a) | 0 (a) | 3 (ab) | 8 (abcd) | 0 (a) | 0 (a) | 2 (ab) | 0 (a) | 1 (a) | 0 (a) | 1 (a) | 1 (a) | 2 (ab) | 0 (a) | 0 (a) | 0 (a) | 0 (a) |
| Serene | 38 (defghij) | 38 (defghij) | 3 (ab) | 0 (a) | 0 (a) | 0 (a) | 0 (a) | 0 (a) | 2 (a) | 4 (abc) | 0 (a) | 0 (a) | 0 (a) | 1 (a) | 1 (a) | 0 (a) | 0 (a) | 0 (a) | 0 (a) | 0 (a) | 0 (a) | 0 (a) | 0 (a) |
| Indifferent | 0 (a) | 10 (ab) | **58 (c)** | 1 (a) | 2 (a) | 8 (ab) | 4 (a) | 4 (a) | 9 (ab) | 7 (ab) | 7 (ab) | 2 (a) | 3 (a) | 3 (a) | 3 (a) | 5 (ab) | 10 (ab) | 7 (ab) | 8 (ab) | 3 (a) | 4 (a) | 4 (a) | 7 (ab) |
| Quiet | 17 (abcdefghi) | 33 (hijk) | 8 (abcd) | 0 (a) | 0 (a) | 1 (a) | 0 (a) | 0 (a) | 2 (ab) | 4 (abc) | 1 (a) | 0 (a) | 0 (a) | 0 (a) | 0 (a) | 0 (a) | 0 (a) | 0 (a) | 0 (a) | 0 (a) | 0 (a) | 0 (a) | 0 (a) |
| Bored | 0 (a) | 0 (a) | 13 (bc) | 3 (ab) | 3 (ab) | 8 (abc) | 13 (bc) | 4 (ab) | 16 (c) | 2 (ab) | 5 (abc) | 2 (ab) | 10 (abc) | 3 (ab) | 2 (ab) | 11 (abc) | 8 (abc) | 16 (c) | 10 (abc) | 5 (abc) | 7 (abc) | 4 (ab) | 7 (abc) |
| Sad | 0 (a) | 0 (a) | 8 (a) | 7 (a) | 8 (a) | 12 (a) | 10 (a) | 5 (a) | 69 (cd) | 2 (a) | 15 (a) | 37 (b) | **75 (d)** | **86 (d)** | 16 (a) | 44 (b) | 52 (bc) | 48 (b) | 5 (a) | 9 (a) | 7 (a) | 7 (a) | 10 (a) |
| Melancholic | 0 (a) | 0 (a) | 11 (abcd) | 13 (abcdef) | 5 (abc) | 10 (abcd) | 10 (abcd) | 4 (abc) | 21 (cdefgh) | 3 (ab) | 23 (defghi) | 28 (efghij) | 38 (ij) | 34 (hij) | 19 (bcdefgh) | 30 (ghij) | 29 (fghij) | **40 (j)** | 12 (abcde) | 15 (abcdefg) | 21 (cdefgh) | 12 (abcde) | 23 (defghi) |
| Unhappy | 0 (a) | 0 (a) | 15 (abcd) | 16 (abcd) | 13 (abc) | 15 (abcd) | 14 (abc) | 14 (abc) | **54 (ghij)** | 3 (ab) | 21 (bcde) | 37 (efgh) | **61 (ij)** | **66 (j)** | 13 (abc) | **46 (fghi)** | **49 (ghij)** | **55 (hij)** | 19 (abcde) | 35 (defg) | 37 (efgh) | 24 (cde) | 28 (cdef) |
| Dissatisfied | 1 (a) | 1 (a) | 16 (abcde) | 14 (abcd) | 12 (ab) | 19 (abcde) | 13 (abc) | 13 (abc) | 46 (f) | 3 (a) | 26 (bcde) | 28 (bcdef) | 25 (bcde) | 25 (bcde) | 12 (ab) | 34 (ef) | 33 (def) | 35 (ef) | 29 (bcdef) | 33 (def) | 30 (bcdef) | 35 (ef) | 32 (cdef) |
| Disappointed | 0 (a) | 0 (a) | 17 (abcde) | 7 (ab) | 8 (ab) | 10 (abc) | 13 (abcd) | 10 (abc) | 51 (h) | 9 (ab) | 24 (bcdef) | 34 (efgh) | 47 (gh) | 39 (fgh) | 10 (abc) | 36 (efgh) | **46 (gh)** | 40 (fgh) | 29 (defg) | 33 (efgh) | 25 (bcdef) | 28 (cdefg) | 30 (defg) |
| Annoyed | 0 (a) | 0 (a) | 11 (abcd) | 24 (cdef) | 22 (cdef) | 24 (cdef) | 15 (abcd) | 24 (cdef) | 11 (abcd) | 5 (abc) | 13 (abcd) | 15 (abcd) | 9 (abcd) | 8 (abcd) | 8 (abcd) | 40 (fg) | 26 (def) | 21 (bcde) | **50 (g)** | 38 (efg) | **47 (g)** | **53 (g)** | 37 (efg) |
| Disgusted | 0 (a) | 0 (a) | 7 (ab) | **87 (d)** | **84 (d)** | 7 (ab) | 9 (ab) | 7 (ab) | 3 (ab) | 2 (ab) | 4 (ab) | 7 (ab) | 4 (ab) | 3 (ab) | 4 (ab) | **44 (c)** | 12 (ab) | 15 (ab) | 17 (b) | 11 (ab) | 11 (ab) | 16 (ab) | 14 (ab) |
| Angry | 0 (a) | 0 (a) | 1 (a) | 0 (a) | 5 (ab) | 3 (ab) | 7 (ab) | 10 (ab) | 2 (ab) | 1 (a) | 1 (a) | 0 (a) | 0 (a) | 2 (ab) | 2 (ab) | 21 (b) | 7 (ab) | 5 (ab) | **74 (c)** | **95 (d)** | **84 (cd)** | **91 (cd)** | **86 (cd)** |
| Worried | 0 (a) | 1 (ab) | 16 (abcde) | 14 (abcd) | 9 (abc) | 32 (defgh) | 40 (ghij) | 32 (defgh) | 19 (abcdef) | 33 (defgh) | **57 (j)** | 53 (ij) | 22 (cdefg) | 20 (bcdef) | **48 (hij)** | 32 (defgh) | 35 (efghi) | 37 (fghi) | 5 (abc) | 5 (abc) | 7 (abc) | 5 (abc) | 8 (abc) |
| Guilty | 0 (a) | 0 (a) | 9 (ab) | 5 (a) | 2 (a) | 10 (abc) | 13 (abc) | 7 (a) | 25 (cde) | 7 (a) | 23 (bcd) | 35 (def) | 32 (def) | 35 (def) | 15 (abc) | 33 (def) | **47 (f)** | 40 (ef) | 3 (a) | 3 (a) | 3 (a) | 2 (a) | 13 (abc) |
| Additional Word | festive (22%) | feeling cool (11%) | - | urge to vomit/ nauseated (12%) | feeling sick (12%) | tired (27%), feeling hot (20%) | dead/deceased (26%) | - | - | - | - | - | - | - | scared/ frightened (22%) | - | - | - | - | - | - | - | - |
